# SARS‐CoV‐2 and lung injury: Dysregulation of immune response but not hyperimmune response as in “cytokine storm syndrome”

**DOI:** 10.1111/crj.13455

**Published:** 2021-11-03

**Authors:** Serpil Öcal

**Affiliations:** ^1^ Department of Internal Medicine, Medical Intensive Care Unit Hacettepe University Faculty of Medicine Ankara Turkey

**Keywords:** ALI, ARDS, critically ill patients, dysregulation of immune response, SARS‐CoV‐2

## Abstract

SARS‐CoV‐2 infection can present either an asymptomatic or symptomatic; the spectrum of symptomatic infection ranges from mild to critical. A majority of patients have experienced mild symptoms with a good prognosis. But approximately 14% of them have severe infection presenting with hypoxemia and extensive lung involvement. The current mini‐review describes the dysregulation of immune response for SARS‐CoV‐2 viral pneumonia and virus‐induced lung injury. Also, many confounding factors can increase lung injury, in addition to virus‐induced lung injury. Especially in critically ill patients, confounding factors can cause the inflammatory cascade, acute respiratory distress syndrome (ARDS), and mortality.

## 1 INTRODUCTION

Many SARS‐CoV‐2 patients have mild symptoms which is typically a self‐limiting upper respiratory disease, but some patients may have viral pneumonia.^1^ Uncommonly, viral pneumonia is not self‐limiting, so severe disease develops. Why is it self‐limiting in some patients and not self‐limiting in others? Recent studies have shown that increased serum levels of inflammatory markers (including C‐reactive protein, ferritin, and D‐dimer), cytokines, chemokines, and an increased neutrophil‐to‐lymphocyte ratio have been associated with disease severity and mortality.^2^ In the light of immunological studies, dysregulation of immune response at the lung may contribute to disease severity, not hyperimmune response “cytokine storm syndrome” (CSS). Also, many confounding factors can increase lung injury in addition to virus‐induced lung injury.

In immunomodulatory therapy studies for SARS‐CoV‐2, there is no clinical definition of CSS. The concept of CSS is a group of disorders with fulminant systemic inflammation and presented with pancytopenia, fever, hepatosplenomegaly, lymphadenopathy, rash, and central nervous system inflammation. The consequent outcome is hemodynamic instability, multiple organ dysfunction, and mortality. CSS clinical phenotypes are as follows: (i) *haemophagocytic lymphohistiocytosis (so‐called macrophage activation syndrome [MAS] in the course of rheumatic diseases)*, (ii) *auto‐inflammatory syndromes*, and (iii) chimeric antigen receptor‐T (CAR‐T) *therapy*.^3^ Although there are not yet standard diagnostic criteria for CSS in patients with SARS‐CoV‐2, studies articulate hyperimmune CSS response as the main immunopathology of the devastating disease. Clinical findings such as pancytopenia, hepatosplenomegaly, lymphadenopathy, rash, and central nervous system inflammation are not seen in critically ill patients with SARS‐CoV‐2 from my experience.

It is very well known that SARS‐CoV‐2 can induce lymphopenia. In severe disease, lymphocyte, CD4^+^ T lymphocyte, and CD8^+^ T lymphocyte counts are negatively correlated with nasopharyngeal SARS‐CoV‐2 RNA load.^4^ Evidence from severe patients with a lower number of lymphocytes reveals the role of dysregulation of immune response in the pathogenesis of COVID‐19.^5^, ^6^ Recent studies found dysregulation of several immune cell types in COVID‐19 related hospitalized patients, whereas most had returned to baseline in nonhospitalized individuals. Wen et al. found increased frequencies of the classical CD14^+^ monocyte population in a small cohort of COVID‐19 recovered patients.^7^ Dijiv et al. reported increased frequencies of “non‐T/non‐B” cells in patients with COVID‐19.^8^ CD4^+^ and CD8^+^ memory T cells are typically generated after the initial activation and expansion stage that occurs in acute infection. As previously demonstrated, the establishment of airway memory CD4^+^ T cells mediated protective immunity against respiratory coronaviruses, including SARS and MERS.^9^ The sustained expression of T cell activation and exhaustion markers in nonhospitalized, convalescent individuals with SARS‐CoV‐2, as well as decreased frequencies of CD27‐ and CD28‐expressing CD8^+^ T cells were reported.^10^ These findings may represent an impaired ability to form memory T cells. This is supported by prior observations concerning IL‐10 production in SARS and SARS‐CoV‐2. Also, IL‐10 production by regulatory CD4^+^ T cells is necessary for memory CD8^+^ T cell development.^11^

In patients with SARS, there was an increase in IL‐10 production during the convalescent phase of infection. A possible etiology for how the sustained immune dysfunction observed during convalescence could impair the formation of long‐term memory T cells, emphasizing the need to explore memory and regulatory T cell development and function in SARS‐CoV‐2 infected individuals. There was substantial B cell activation demonstrated by increased frequencies of CD95^+^, CD69^+^, and PD1^+^ B cells in hospitalized group.^12^ This may reflect both the presence of SARS‐CoV‐2‐specific B cells responding to antigen and/or bystander B cell activation. B cell markers were found to generally return to levels similar to healthy controls, although the sustained presence of FCRL4^+^ and PD1^+^ B cells suggests persistence of some degree of B cell dysregulation.^12^ How this dysregulation relates to SARS‐CoV‐2 antibody responses is unknown. The finding of more pronounced T cell activation/exhaustion in elderly nonhospitalized individuals with SARS‐CoV‐2 has many potential implications. In acute disease, these findings suggest that this group may be at heightened risk for inflammation‐mediated pathology. This immune dysfunction may also lead to suboptimal SARS‐CoV‐2‐specific memory responses and increased susceptibility to reinfection.

There are complex feedback interactions among the cytokines that are involved in inflammatory reactions. Critically ill patients have increased serum levels of IL‐2, IL‐6, IL‐7, IL‐10, macrophage colony‐stimulating factor (M‐CSF), granulocyte colony‐stimulating factor (G‐CSF), granulocyte‐macrophage colony‐stimulating factor (GM‐CSF), 10 kD interferon‐gamma‐induced protein (IP‐10), monocyte chemoattractant protein‐1 (MCP‐1), macrophage inflammatory protein 1‐α (MIP 1‐α), and TNF‐α.^13^, ^14^ Both pro‐inflammatory cytokines (IL‐6, IL‐8, and TNF‐α) and anti‐inflammatory cytokines (IL‐10) co‐exist in the circulation of critically ill patients. None of these cytokines were overproduced as in CSS. Once an inflammatory reaction is started, a single factor like IL‐6 cannot terminate the immune response of the entire network. Inappropriate immune intervention, especially for infections of critically ill patients, may further destroy the immune equilibrium.

IL‐6 is the most studied inflammatory marker in patients with SARS‐CoV‐2; slow temporal changes of IL‐6 serum level in patients with severe disease with SARS‐CoV‐2 are observed, not peak changes of it as in CSS.^5^, ^13^, ^15^ Also, at peak changes in CSS, serum levels of IL‐6 showed >1000 pg/ml, while it was reported <1000 pg/ml in severe patients with SARS‐CoV‐2. Sayah et al. reported that the median serum level of IL‐6 was 114 pg/ml in severe disease without steroid therapy. It was the most accurate inflammatory biomarker observed, and the calculated cut‐off for serum level of IL‐6 (42 pg/ml) could correctly classify *>*90% of patients regarding their risk of severity (AUROC = 0.972).^15^ Furthermore, most critically ill patients are older and have more underlying diseases including hypertension and diabetes.^13^, ^14^ Also, the serum levels of IL‐6 were significantly higher in patients with comorbidities without viral infection.^16^

Most researchers have failed to identify too high serum levels of IL‐6 (>1000 pg/ml); instead, many observed variable serum levels of IL‐6 which point out dysregulation of immune response. The preceding concept has been confirmed in three randomized double‐blind, placebo‐controlled trials (COVACTA, EMPACTA, and BACC bay); antagonistic IL‐6 treatments have not shown definitive success in improving patient outcome.^17^, ^18^, ^19^ In contrast, REMAP‐CAP and RECOVERY trials demonstrated mortality benefit from treatment with tociuzumab.^20^, ^21^ In clinical practice, tocilizumab treatment in critically ill patients complicates patient follow‐up due to inhibition of the inflammatory markers including CRP, ferritin, and D‐dimer. In my opinion, PaO_2_/FiO_2_ or SpO2/FiO_2_ remains to be the only decision‐making tool at clinical follow‐up. Although the extent of lung involvement can be evaluated via computed tomography (CT) scan, its overuse during the COVID‐19 pandemic raises concerns about radiation‐induced adverse health effects, both in patients and in healthcare workers.

During this global COVID‐19 pandemic, steroids are the most commonly used immunomodulators therapy. It is very well known that these drugs are also immunosuppressive drugs and enhance viral replication in primary human airway cells. Both in vitro and in vivo studies demonstrate that it enhances virus replication in the respiratory tract and impairs type‐I interferon's antiviral response.^22^ In addition, the inhaled steroids are associated with an increased risk of secondary infections, particularly pneumonia in patients with asthma and exacerbations of COPD.^23^ In critically ill patients at higher risk of secondary infections, steroid‐related alterations of hemogram remain to be another problem: In presence of elevated leukocytes or neutrophils, the suspicion arises whether if it is the sign of a secondary infection or the drug effect.

Several infectious or noninfectious factors are associated with impaired oxygenation and lung injury in severe patients other than viral‐lung injury. Confounding factors including infectious or noninfectious factors should be minimized to limit the inflammatory cascade. These factors can be summarized as follows:

- Firstly, oxygen toxicity (FiO_2_ ≥ 60% exposure more than 6–8 h) increases lung injury, so hypoxemia should be treated promptly and SpO2 target should be achieved by avoiding oxygen toxicity. Also, pulmonary oxygen toxicity can result in lung recovery by fibrosis.^24^
- Spontaneous breathing offers physiological benefits such as preserved diaphragm activity, improved cardiovascular functions, and reduced need for sedation. However, negative transpulmonary pressure may increase during spontaneous breathing in the lung with low compliance—a stiff lung—so the high respiratory drive of excessive breathing‐effort that might lead to self‐inflicted lung injury.
- Patient‐ventilator asynchrony during mechanical ventilation or withdrawal syndrome to sedoanalgesic drugs during weaning may impair the oxygenation of the patient and increase lung injury and cause the inflammatory cascade if it gets longer.
- Barotrauma due to too high PEEP and/or tidal volumes by mechanical ventilatory increases lung injury. In critically ill patients applied invasive mechanical ventilation, Pplato and Pdriving should be checked to prevent baratrauma.
- Frequent and long‐time aggressive endotracheal suctioning (to remove secretion) resulting in functional residual capacity reduction increases lung injury during mechanical ventilation.
- Inappropriate intravenous fluid therapy and hypervolemia should be avoided in acute lung injury (ALI)/ acute respiratory distress syndrome (ARDS). Intravenous fluid therapy increases interstitial lung edema, so impairs the oxygenation of the lung. Keeping the patient normovolemic (if necessary administering diuretics) should be the mainstay of fluid therapy. Notably, vasopressors might be considered when the diuretic effects to resolve interstitial edema are delayed.
- Coinfections with bacterial pathogens in patients with viral pneumonia have been well documented for the 1918–1919 influenza pandemic.^25^ Clinical variables, such as patient characteristics, chest radiographic findings, or routine laboratory results‐especially tocilizumab, and glucocorticosteroid, were used and are unreliable for distinguishing between viral and bacterial pneumonia. Low procalcitonin levels may not rule out secondary bacterial infections in patients with extensive lung injury.

Unfortunately, the immunologic mechanisms leading to ALI/ARDS in patients with SARS‐CoV‐2 and other respiratory viruses are incompletely understood. Lung injury caused by dysregulation of immune response to SARS‐CoV‐2 may progress with an inflammatory cascade triggered by confounding factors. Clinical confounding factors should be considered in immunopathogenesis studies.

## FUNDING INFORMATION

None.

## CONFLICT OF INTEREST

I have no conflict of interest with the contents of this article.

## Data Availability

Data sharing is not applicable to this article as no datasets were generated or analyzed during the current study.
